# Tea and coffee drinking and ovarian cancer risk: results from the Netherlands Cohort Study and a meta-analysis

**DOI:** 10.1038/sj.bjc.6604008

**Published:** 2007-10-09

**Authors:** J Steevens, L J Schouten, B A J Verhage, R A Goldbohm, P A van den Brandt

**Affiliations:** 1 Department of Epidemiology, Maastricht University, PO Box 616, 6200 MD Maastricht, The Netherlands; 2 TNO Quality of Life, Leiden, The Netherlands

**Keywords:** tea, coffee, ovarian neoplasms, meta-analysis, aetiology, cohort studies

## Abstract

In a cohort study, ovarian cancer (280 cases) showed no significant association with tea or coffee, the multivariable rate ratios being 0.94 (95% confidence interval (CI): 0.89, 1.00) and 1.04 (95% CI: 0.97, 1.12) per cup per day, respectively. A meta-analysis also produced no significant findings overall, though the cohort studies showed a significant inverse association for tea.

Tea and coffee are widely consumed around the world and may affect human health. Several case-control and cohort studies have analysed the relationship with ovarian cancer risk, yielding inconclusive results. We analysed the association between tea, coffee and ovarian cancer in a prospective cohort study and summarised results of previous studies in a meta-analysis.

## MATERIALS AND METHODS

### The cohort

The Netherlands Cohort Study on Diet and Cancer is a prospective cohort study (*n*=120 852) that started in September 1986 with the enrolment of participants aged 55–69, among whom 62 573 were women (van den Brandt *et al*, 1990). Data processing and analysis were based on the case-cohort approach. A subcohort of 2589 women was randomly drawn from the cohort at baseline. After exclusion of prevalent cancer cases (other than skin cancer) (*n*=151), women who reported at baseline to have undergone an oophorectomy (*n*=32) and subcohort members with incomplete or inconsistent dietary questionnaire data (*n*=190) (Goldbohm *et al*, 1994), incomplete information on tea or coffee consumption (*n*=80) or confounders (*n*=53), 2083 subcohort members were available for analysis.

### Follow-up

The subcohort has been followed up for emigration and vital status. No female subcohort members were lost to follow-up. Follow-up for epithelial ovarian cancer incidence was performed by record linkage to the Netherlands Cancer Registry. During 13.3 years of follow-up, 362 incident, microscopically confirmed ovarian carcinomas (ICD-O-3 code C56.9) were identified. After exclusion of borderline invasive (*n*=14) and non-epithelial tumours (*n*=12) and women with incomplete or inconsistent dietary questionnaire data (*n*=36), missing information on tea or coffee consumption (*n*=11) or confounders (*n*=9), 280 cases remained available for analyses.

### Questionnaire

A self-administered questionnaire on risk factors for cancer, including a food frequency questionnaire (FFQ), was completed by all cohort members at baseline. The respondents were asked whether they drank tea or coffee and, if so, how many cups per day. The type of tea was not specified but this population rarely drank any tea other than black tea in 1986 (Goldbohm *et al*, 1996). We validated the FFQ against a 9-day diet record (Goldbohm *et al*, 1994) and established the 5-year reproducibility of the FFQ (Goldbohm *et al*, 1995).

### Data analysis

Person-years of follow-up were calculated for the subcohort members from the start of the study until the date of ovarian cancer diagnosis, death, emigration or end of follow-up. Incidence rate ratios (RR) and corresponding 95% confidence intervals (CI) were estimated in age-adjusted and multivariable-adjusted analyses using Cox proportional hazards model with Stata 9.0.

### Meta-analysis

We performed a meta-analysis of case-control and cohort studies on tea, coffee and ovarian cancer using the Stata procedure ‘metan’. A random effects model was used because of significant heterogeneity. On tea drinking and ovarian cancer, eight case-control studies (Byers *et al*, 1983; Miller *et al*, 1987; La Vecchia *et al*, 1992; Kuper *et al*, 2000; Tavani *et al*, 2001; Zhang *et al*, 2002; Jordan *et al*, 2004; Baker *et al*, 2007) and, including this study, five cohort studies (Zheng *et al*, 1996; Larsson and Wolk, 2005b; Gates *et al*, 2007; Silvera *et al*, 2007) have been conducted. Coffee drinking and ovarian cancer risk was investigated in 16 case-control studies (Trichopoulos *et al*, 1981; Hartge *et al*, 1982; Byers *et al*, 1983; Cramer *et al*, 1984; La Vecchia *et al*, 1984; Tzonou *et al*, 1984; Miller *et al*, 1987; Whittemore *et al*, 1988; Polychronopoulou *et al*, 1993; Kuper *et al*, 2000; Tavani *et al*, 2001; Jordan *et al*, 2004; Riman *et al*, 2004; Baker *et al*, 2007) and, including this study, five cohort studies (Snowdon and Phillips, 1984; Stensvold and Jacobsen, 1994; Larsson and Wolk, 2005a; Silvera *et al*, 2007). We had to exclude from the meta-analysis studies that did not report 95% CIs (Trichopoulos *et al*, 1981; Byers *et al*, 1983; Cramer *et al*, 1984; Tzonou *et al*, 1984; Stensvold and Jacobsen, 1994).

## RESULTS

After 13.3 years of follow-up, there were 280 epithelial ovarian carcinomas, 47.1% of which were serous carcinomas, 10.0% mucinous carcinomas, 9.3% endometrioid carcinomas and 4.3% clear cell carcinomas. Tea was drunk by >89% of the subjects, coffee by >96% and only 0.2% drank neither. Mean daily tea consumption (s.d.) was 3.1 (2.1) cups for subcohort members and 2.9 (1.8) cups for cases. Older women, never smokers, more highly educated respondents and women with a normal body mass index (BMI) drank more tea. Mean daily coffee consumption (s.d.) was 4.0 (2.0) cups for subcohort members and 4.1 (2.0) cups for cases. A lower age, current smoking, a lower level of education and a higher BMI were associated with higher coffee consumption.

Table 1 shows RRs for ovarian cancer for tea and coffee consumption. Tea consumption was inversely associated with ovarian cancer risk, although not statistically significant and coffee consumption showed no association. The proportional hazards assumption was not violated and there was no effect modification. The category 1–<3 cups per day was chosen as a reference because there were too few subjects in the lowest category. We excluded subjects with <2 years of follow-up to check for protopathic bias, analysed stable coffee drinkers separately and corrected for acrylamide in coffee, but none of these analyses affected the results. Subtype analyses showed that tea was inversely associated with serous tumours, whereas it was related to increased risk of mucinous and endometrioid tumours, giving some indication for heterogeneity.

**Figure 1:**
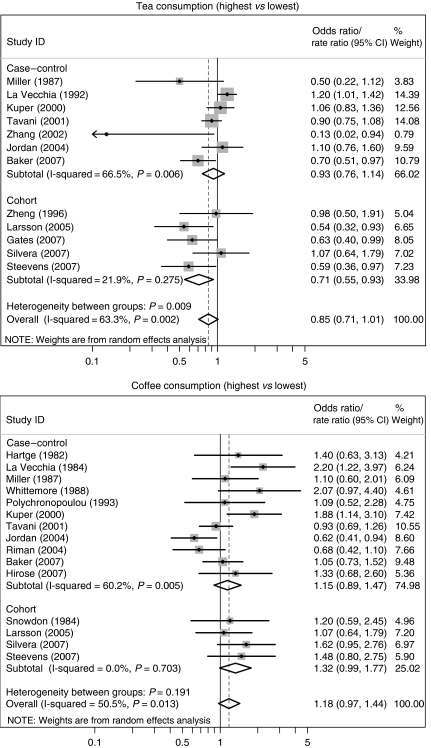
Meta-analysis of case-control and cohort studies investigating tea and coffee consumption (highest *vs* lowest) in relation to ovarian cancer risk. Note: In this meta-analysis, we used the lowest category as a reference in all studies, for reasons of comparability.

In the meta-analysis (Figure 1), tea was found to be inversely associated with ovarian cancer when study results were pooled. The association was strongest, and statistically significant, in cohort studies. Coffee consumption was related to increased ovarian cancer risk when study results were pooled. A stronger, borderline significant association was seen in cohort studies.

## DISCUSSION

In this cohort study, coffee consumption was not associated with the risk of epithelial ovarian cancer in postmenopausal women. Tea drinking was inversely, but not statistically significantly, associated with ovarian cancer risk. An important strength of this study is the prospective character, which makes recall bias unlikely.

The heterogeneity between the outcomes observed in different studies may be attributable to effect modification by genetic factors, for example polymorphisms of the CYP1A2 genotype, an enzyme involved in the metabolism of caffeine (Goodman *et al*, 2003). As tea and coffee are very common beverages, even a modest decrease or increase of ovarian cancer risk found in the meta-analysis is of importance.

## Figures and Tables

**Table 1:** Rate ratios of ovarian cancer according to coffee and tea consumption^a^

				**Age-adjusted**		**Multivariable adjusted^b^**	
	**Mean (cups per day)**	**Cases (*n*)**	**Person- years in subcohort**	**RR**	**95% CI**	**RR**	**95% CI**
*Coffee* (*cups per day*)							
0–<1	0.5	15	1913	0.70	0.39, 1.25	0.73	0.41, 1.31
1–<3	2.5	87	7647	1.00	Reference	1.00	Reference
3–<5	4.3	119	11,243	0.96	0.71, 1.29	1.00	0.74, 1.35
⩾5	6.9	59	5124	1.07	0.75, 1.53	1.08	0.75, 1.57
				*P*-trend=0.33		*P*-trend=0.35	
Coffee increment (1 cup per day)				1.04	0.98, 1.11	1.04	0.97, 1.12
							
*Tea* (*cups per day*)							
0–<1	0.5	66	5455	1.13	0.81, 1.57	1.10	0.78, 1.54
1–<3	2.4	107	9856	1.00	Reference	1.00	Reference
3–<5	4.2	83	7262	1.03	0.76, 1.40	1.04	0.76, 1.42
⩾5	6.8	24	3354	0.64	0.40, 1.02	0.65	0.41, 1.03
				*P*-trend=0.07		*P*-trend=0.12	
Tea increment (1 cup per day)				0.94	0.88, 1.00	0.94	0.89, 1.00

Abbreviations: RR=rate ratio; CI=confidence interval.

a The Netherlands Cohort Study (1986–1999).

b Adjusted for age (years), use of oral contraceptives (ever/never), parity (number of children), cigarette smoking (current, ex-smoker, never smoker). Coffee and tea were mutually adjusted.
